# Effective low-dose Anlotinib induces long-term tumor vascular normalization and improves anti-PD-1 therapy

**DOI:** 10.3389/fimmu.2022.937924

**Published:** 2022-08-03

**Authors:** Peng Fan, Huiping Qiang, Zhenhua Liu, Qi Zhao, Ying Wang, Tingkun Liu, Xuan Wang, Tianqing Chu, Yuhui Huang, Wei Xu, Songbing Qin

**Affiliations:** ^1^ Cyrus Tang Hematology Center, Collaborative Innovation Center of Hematology, State Key Laboratory of Radiation Medicine and Prevention, Soochow University, Suzhou, China; ^2^ Department of Experimental Medicine, University of Rome “Tor Vergata”, Rome, Italy; ^3^ Department of Respiratory Medicine, Shanghai Chest Hospital, Shanghai Jiao Tong University, Shanghai, China; ^4^ Department of Radiotherapy, The First Affiliated Hospital of Soochow University, Suzhou, China; ^5^ Department of Immunology, Innovent Biologics, Inc., Suzhou, China

**Keywords:** Effective low-dose, Anlotinib, Anti-PD1 therapy, CD8+ T cell, Long-term vascular normalization

## Abstract

Anlotinib is a new multitarget tyrosine kinase inhibitor for tumor angiogenesis, and its monotherapy exhibits a decent clinical efficacy. However, the process of combining Anlotinib and immune checkpoint therapy to achieve optimal antitumor effects while limiting side effects remains unclear. In this study, we found that effective low-dose Anlotinib was sufficient to inhibit tumor growth while reducing side effects compared with high doses. Effective low-dose Anlotinib treatments induced durable tumor vascular normalization and improved anti-PD-1 therapy in both short- and long-term treatment regimens. Mechanistically, the combination therapy increased the proportions of intratumoral CD4^+^ T, CD8^+^ T, and NK cells. Anlotinib-associated antitumor effects were independent of interferon γ; however, the combination therapy required CD8^+^ T cells to suppress tumor growth. Together, these results suggest that the combination of effective low-dose Anlotinib and PD-1 blockade induces durable antitumor effects with fewer side effects. Our findings indicate that antiangiogenic treatments combined with immune checkpoint therapy at an effective low-dose, rather than a tolerable high dose, would be more efficacious and safer.

## Introduction

Angiogenesis, or the formation of new blood vessels from existing blood vessels, is essential for tumor initiation and progression (1, 2). Antiangiogenic therapy is widely used in the clinical treatment of various solid tumors. However, the development of diverse resistance mechanisms lessens the therapeutic effects of antiangiogenic treatments (3–7). The most common proposed resistant mechanism is related to increased tumor tissue hypoxia caused by antiangiogenic therapy, which elevates hypoxia-inducible factor 1α (HIF1α), thereby inducing or upregulating alternate proangiogenic growth factors (3, 4, 8). Emerging clinical data suggest that patients whose tumor perfusion or oxygenation increases in response to antiangiogenic therapy may actually survive longer (9–13). In addition, tumor hypoxia is also an important impediment to many effective cancer therapies. For example, the primary cytotoxicity of radiotherapy is the creation of reactive oxygen species (ROS), thus hypoxic tumors are usually resistant to radiotherapy. A number of chemotherapeutic drugs have been shown to be less effective when exposed to a hypoxic environment, which can lead to further disease progression (14, 15). Hypoxia is also a potent barrier to effective immunotherapy in cancer treatment (16). Therefore, strategies to utilize antiangiogenic agents to improve tumor blood vessel perfusion and alleviate hypoxia may enhance the therapeutic outcomes of concurrent radiotherapy, chemotherapy, or immunotherapy.

Several lines of evidence have indicated that antiangiogenic therapy can normalize the tumor vasculature and alleviate hypoxia (17, 18). However, the window of vascular normalization induced by antiangiogenic therapy is often transient (9, 19). Antiangiogenic therapy is generally used at a high dose, which may cause excessive pruning of tumor vessels and thus shorten the normalization window, resulting in the induction of resistance and the impairment of concurrent therapeutic agents (9, 20). Moreover, high doses and long-term drug administration can cause more side effects, such as hypertension, proteinuria, thromboembolism, hemorrhage, fistula formation, and bowel perforation (21, 22), which may interrupt treatments and worsen the quality of life of cancer patients. Hence, it is expected that the dosage and frequency of antiangiogenic treatments may influence the normalizing effects and subsequent therapeutic efficacy, especially in combination therapy.

Anlotinib, a multitargeted tyrosine kinase inhibitor mainly targeting vascular endothelial growth factor receptor (VEGFR), fibroblast growth factor receptor (FGFR), platelet-derived growth factor receptors (PDGFR), and c-kit, has been approved for the third-line treatment of non-small cell lung cancer (NSCLC) and second-line treatment of soft tissue sarcoma in China (23, 24). However, tumor control by Anlotinib alone remains limited. The combination of Anlotinib and anti-PD-1 therapy has been shown to improve antitumor responses (25, 26). A preclinical study indicated that Anlotinib could transiently normalize the tumor vasculature and improve the therapeutic effect of PD-1 blockade (27). The clinical data of anti-PD-1 treatment concomitant with Anlotinib also showed enhanced antitumor activity in patients with various advanced tumors (28–30). However, the process of coordinating Anlotinib and PD‐1 blockade therapy to maximize the beneficial effects while reducing adverse influences remains unclear. Here, we investigated the impacts of the dosage and duration of Anlotinib on the tumor microenvironment and anti-PD-1 therapy in an orthotopic breast tumor model. Our data showed that effective low-dose Anlotinib reconditioned the tumor immune microenvironment and enhanced anti-PD-1 therapy in both short- and long-term treatment regimens while reducing side effects.

## Materials and methods

### Mice

Female C57BL/6 mice were ordered from the Shanghai SLAC Laboratory Animal Center (Shanghai, China). FVB mice were bred and maintained in the specific pathogen-free (SPF) animal facility at Soochow University. *Ifnγ^−/−^* C57BL/6 mice were a generous gift from Dr. Jinping Zhang (Soochow University). Female mice (6–8 weeks) were used in all experiments. All mice were bred and housed under SPF conditions in the animal facility at Soochow University.

### Cell lines and reagents

The EO771 murine mammary tumor cell line was obtained from the CH3 Biosystems (Amherst, NY, USA). The lung carcinoma cell line LAP0297 is a generous gift from Dr. Peigen Huang (Massachusetts General Hospital, MA, USA). Tumor cells were cultured at 37°C in a humid incubator containing 5% CO_2_ in Dulbecco’s modified Eagle’s medium (Gibco, USA) supplemented with 10% heat-inactivated fetal bovine serum (Gibco, USA) and 1% penicillin and streptomycin (Gibco, USA). Cells were regularly checked for mycoplasma contamination. Anlotinib, a gift from Jiangsu Chia-Tai Tianqing Pharmaceutical Co. Ltd. (Nanjing, China), was dissolved with sterile ddH_2_O to a final concentration of 10 mg/ml. The stock solution was kept away from the light and stored at 4°C. Anti-mouse PD-1 antibody (Lot number 190312007) was provided by Innovent Biologics Co. Ltd. (Suzhou, China), and anti-CD8a monoclonal antibody (Clone 53-6.72), anti-CD4 monoclonal antibody (Clone GK1.5), or their isotype-matched control antibody IgG2a (2A3) were purchased from Bio X Cell (New Hampshire, USA).

### Tumor growth and treatments

EO771 mammary tumor cells (2 × 10^5^ cells) were orthotopically inoculated into the third mammary fat pad of C56BL/f female mice. Anlotinib or ddH_2_O was administrated by oral gavage once a day. The anti-PD-1 antibody or isotype-matched IgG2a was intraperitoneally injected at a dose of 5 mg/kg every 3 days for the duration of the experiment. Female FVB mice were subcutaneously injected with 5 × 10^4^ LAP0297 lung tumor cells. When LAP0297 lung tumors reached approximately 3 × 4 mm in diameter, mice were randomly grouped and administered with ddH_2_O or Anlotinib (4 mg/kg) daily by oral gavage or anti-PD-1 as well as isotype IgG (2.5 mg/kg) *via* the tail vein every 3 days. The tumor size was monitored closely and measured every 3 days using a caliper, and the tumor volume was estimated by the following formula: (long axis) × (short axis)^2^× π/6. The tumor weight was measured at the end of the experiments.

*In vivo* depletion of CD4^+^ or CD8^+^ T cells was conducted according to our previously published approach (31). Briefly, on days 5, 7, and 12 following EO771 breast tumor cell inoculation, mice were injected intraperitoneally with 200 µg of anti-CD8a monoclonal antibody and anti-CD4 monoclonal antibody or 200 µg of isotype-matched control antibody IgG2a. Flow cytometry was used to determine the effectiveness of T-cell depletion at the end of the experiments.

### Tumor vessel perfusion analysis

Tumor blood perfusion was analyzed based on our previously published procedure (32). Briefly, mice were systemically perfused with PBS after receiving an intravenous injection with 200 µl of PBS solution containing 10 mg/kg of Hoechst 33342 (Sigma, USA) for 5 min. Tumors were then excised and fixed with 4% paraformaldehyde (PFA) for 2–3 h. The perfused blood vessels throughout the tumor tissues were stained with fluorescent nucleus-bound Hoechst 33342 by this approach. To counterstain the slides, nonspecific nuclear staining Sytox Green (Catalog S7020, Molecular Probes, USA) was used. Images were captured using a confocal laser scanning microscope (Olympus, FV3000, Japan). The mean fluorescence intensity of Hoechst 33342 in each field was determined by using Image-Pro Plus software (version 6.0).

### Immunohistochemistry and image analysis

Tumor blood vessel staining and analysis were carried out as reported previously (31). Briefly, tumor tissues were fixed in 4% PFA at 4°C for 3 h and then incubated with 30% sucrose overnight at 4°C. After that, the tissues were embedded in the optical coherence tomography (OCT) compound and stored at −80°C. Frozen samples were sectioned at a 20-µm thickness. The tumor tissue slices were incubated overnight with primary antibodies (anti-CD31, 1:200, clone MEC13.3, Catalog 550274, BD Biosciences, USA) at 4°C and subsequently with the secondary antibody (Alexa Fluor 647 Goat Anti-Armenian Hamster, 1:200, Catalog 127-605-160, Jackson ImmunoResearch, USA) for 2 h at room temperature. As to PD-L1 staining, the tumor tissue slices were incubated with Alexa Fluor 488 anti-PD-L1 (1:100, clone MIH5, Catalog 2212830, Invitrogen, USA) for 2 h at room temperature. Incubations were performed in humid chambers in the dark. The slices were counter-stained with Sytox Green. Sections were imaged with an Olympus FV3000 confocal laser-scanning microscope. The tumor vessel density was calculated using Image-Pro Plus software (version 6.0).

### Flow cytometric analysis

Flow cytometric analysis was performed as described previously (25). Briefly, tumor tissues were cut into pieces and digested with DMEM containing hyaluronidase (1.5 mg/ml, Sigma-Aldrich, USA), collagenase type 1A (1.5 mg/ml, Sigma-Aldrich, USA), and deoxyribonuclease I (20 U/ml, Sigma-Aldrich, USA) at 37°C for 45 min. The tissue mixture was passed through a 70-µm nylon cell strainer to make single cell suspension, which was then washed and resuspended in cold flow buffer (1% bovine serum albumin and 0.1% NaN_3_ in PBS). After being blocked with a rat anti-mouse CD16/CD32 antibody (BD Pharmingen, USA), the following fluorochrome-conjugated anti-mouse antibodies were used: CD45-BV421, CD11b-BV510, CD8a-PE-Cy7, CD4-PE, CD25-APC, NK1.1-APC-Cy7, F4/80-FITC, CD206-PE-Cy7, CD11c-APC, and Gr-1-APC-Cy7 (all from BioLegend, USA). 7-Amino-actinomycin D (7AAD) (eBioscience, USA) was used as a viability dye to exclude dead cells. Flow cytometric data were collected using a Gallios flow cytometer (Beckman, USA) and analyzed using the Kaluza software (version 1.3).

### Statistical analysis

Statistical analyses were conducted using Prism GraphPad (version 9). Experimental differences were determined by unpaired Student’s *t*-test (two-tailed) when comparing two independent groups, and one‐way ANOVA was used to compare more than two groups. The quantitative data are presented as the mean ± standard error of the mean (SEM). Differences were considered statistically significant when *p* < 0.05.

## Results

### Relatively low-dose Anlotinib monotherapy is sufficient for its antitumor effects and superior to inducing tumor vascular normalization

**Figure 1:**
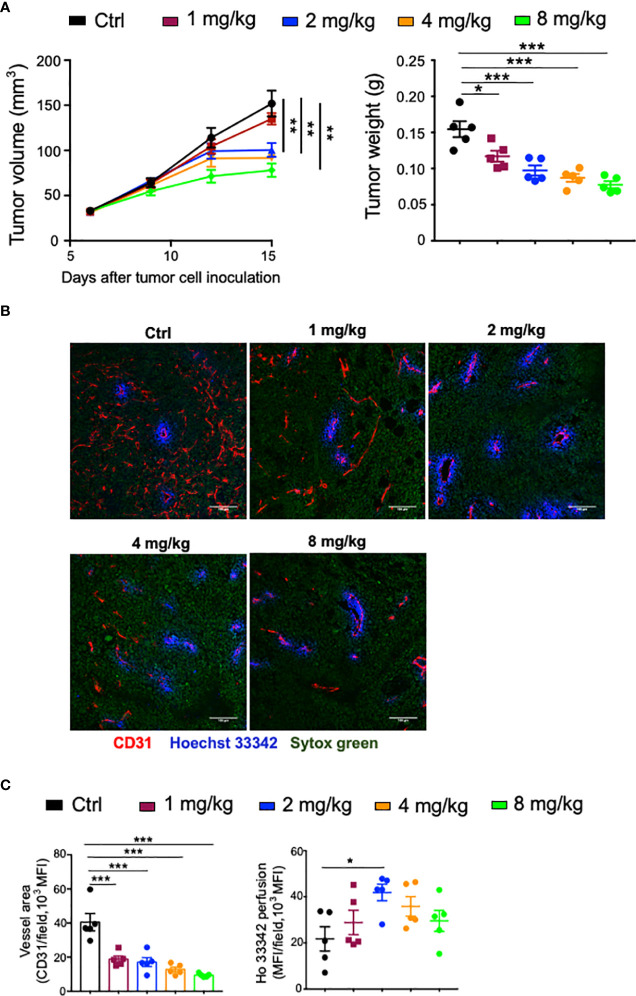
The dose effects of Anlotinib on tumor growth and vascular normalization in orthotopic EO771 breast tumor model. Female C57BL/6 mice were orthotopically inoculated with 2 × 10^5^ EO771 breast tumor cells. When tumors reached 3–4 mm in diameter, mice were randomly grouped and administered with ddH_2_O or different doses of Anlotinib daily by oral gavage. Five minutes prior to tumor harvest, mice were intravenously injected with 200 µg/mouse Hoechst 33342 (Ho33342). Tumor size was recorded every 3 days, and tumor weight was measured at the end of the experiments. **(A)** Tumor growth curves and tumor weight. **(B)** Representative images of Ho33342 perfusion (blue) and CD31-staining (red). Scale bar = 100 µm. **(C)** Ho33342 perfused tumor areas and vessel density (CD31). Significance was determined by one-way ANOVA (*n* = 5 mice per group). Data are presented as mean ± SEM. ^*^ *p* < 0.05; ^**^ *p* < 0.01; ^***^ *p* < 0.001.

As a multitargeted tyrosine kinase inhibitor mainly targeting proangiogenic signaling pathways, the dosage of Anlotinib may influence its outcomes. Therefore, the effects of the dosage of Anlotinib treatments on tumor growth were tested in an orthotopic breast tumor model. We treated EO771 tumor-bearing mice with 1, 2, 4, or 8 mg/kg Anlotinib daily for 9 days. All tested dosages of Anlotinib significantly inhibited EO771 tumor growth compared with the vehicle control group (**Figure 1A**). Anlotinib at a dosage of 2 mg/kg (relatively low-dose) showed significantly better tumor growth inhibition compared with that of 1 mg/kg, while Anlotinib at the dosages of 2, 4 (relatively medium dose), or 8 mg/kg (relatively high dose) had similar antitumor effects (**Figure 1A**). The data show that a relatively low-dose Anlotinib is sufficient to fulfill its antitumor effects. We then analyzed the effects of Anlotinib treatments on tumor angiogenesis. After 9 days of Anlotinib treatments, we harvested EO771 breast tumor tissues and analyzed their tumor blood vessels. Anlotinib treatments at the dosage of 1 mg/kg/daily significantly reduced tumor blood vessel density compared with the control group (**Figures 1B, C**). Anlotinib treatments at a higher dosage, from 2 to 8 mg/kg/daily, showed similar blood vessel density reduction, compared with 1 mg/kg/daily (**Figures 1B, C**). The data suggest that a relatively low-dose Anlotinib is sufficient to accomplish its antiangiogenic effects. Besides tumor blood vessel density, vessel function is another critical parameter to reflect the impacts of Anlotinib treatments on tumor blood vessels. Therefore, we injected Hochest 33342 dye *via* tail vein 5 min before tumor tissue harvest to label functional tumor blood vessels as well as the vessel’s perfused area. Anlotinib treatments at the dosage of 2 mg/kg/daily, but not other dosages, improved tumor blood vessel perfusion compared with the control group (**Figures 1B, C**). In addition, Anlotinib treatments reduced vessel tortuosity and branches compared with the control group (**Figure 1B**). Together, the data suggest that relatively low-dose Anlotinib treatments are sufficient to achieve maximum antitumor effects and superior to inducing tumor vascular normalization in a monotherapy setting. Thus, we named this “relative low-dose” as “effective low-dose” for the rest of the study.

### Effective low-dose Anlotinib treatments improve anti-PD-1 therapy in both short- and long-term treatment regimens with reduced side effects

**Figure 2:**
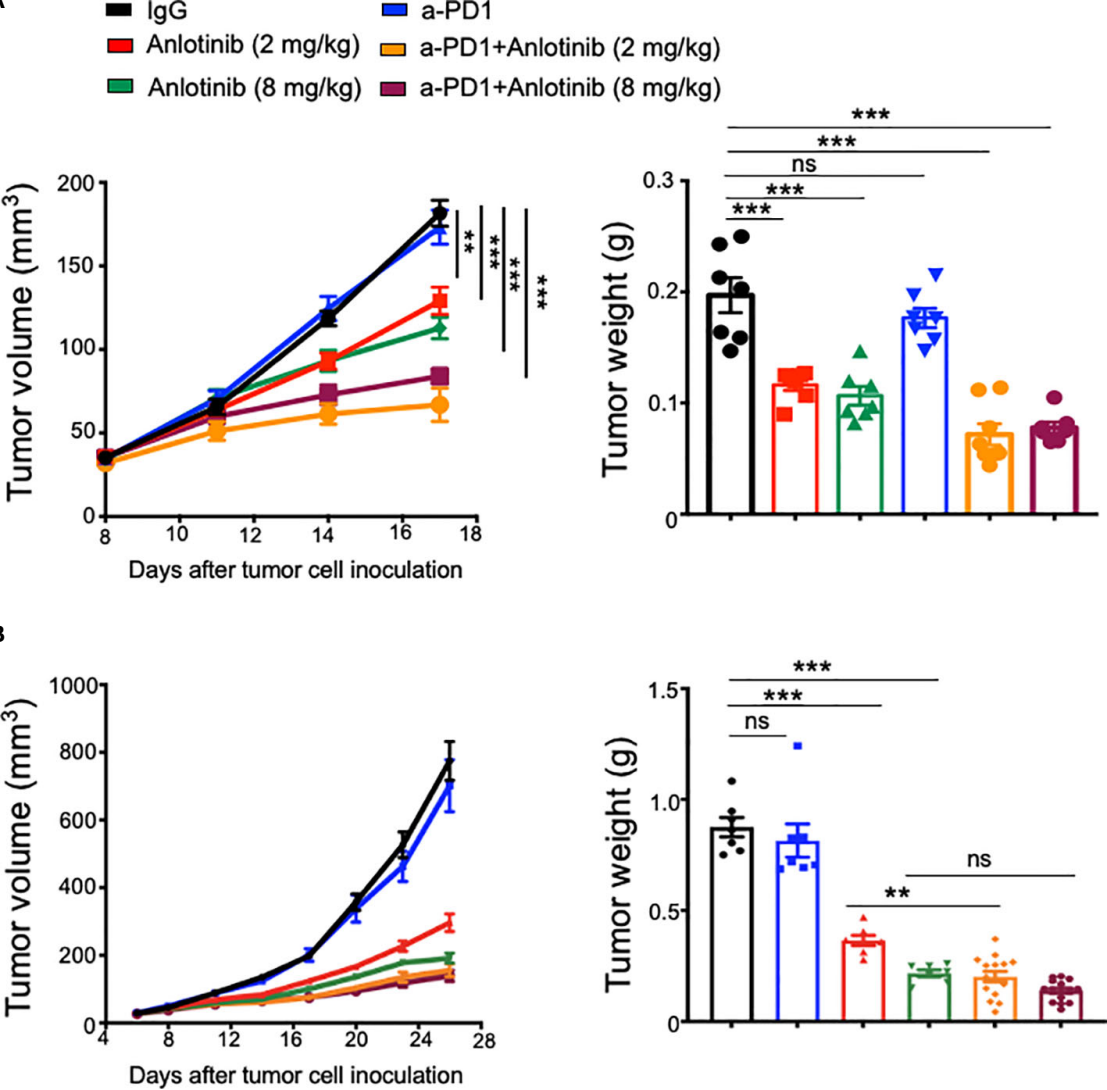
Effective low-dose Anlotinib improved anti-PD-1 therapy in both short- and long-term treatment regimens. EO771 tumor-bearing mice were prepared, and Anlotinib treatments (2 or 8 mg/kg/daily) were conducted as described in **Figure 1**. Treatments with anti-PD-1 or isotype IgG (5 mg/kg) were initiated when tumors reached 3–4 mm in diameter and continued every 3 days. Tumor growth curves and tumor weights with short-term **(A)** or long-term **(B)** combinations of Anlotinib and anti-PD-1 therapy are shown. The significance was determined by one-way ANOVA (*n* = 7–15 mice per group). The data are presented as means ± SEM. NS, no significant difference; ^**^ *p* < 0.01; ^***^ *p* < 0.001.

Recent clinical studies suggest that antiangiogenic therapy can improve immune checkpoint therapy (8, 33–36). To investigate the influence of Anlotinib on immune checkpoint therapy, we combined anti-PD-1 therapy with either a low or high dose of Anlotinib in an orthotopic EO771 breast tumor model. Anlotinib at 2 or 8 mg/kg/daily significantly inhibited EO771 breast tumor growth, compared with the vehicle control group (**Figure 2A**). Although anti-PD-1 therapy alone did not affect EO771 tumor growth, the combination of anti-PD-1 therapy with either 2 or 8 mg/kg Anlotinib treatments significantly enhanced tumor growth inhibition (**Figure 2A**).

To test whether the improvement of low-dose Anlotinib treatments on anti-PD1 therapy can be obtained in another tumor model, we conducted similar combination therapy in the LAP0297 lung tumor model. Firstly, we treated LAP0297 lung tumor-bearing mice with different doses of Anlotinib. Anlotinib at the dosages of 4 mg/kg (relatively low-dose), 8 mg/kg (relatively medium dose), or 16 mg/kg (relatively high dose) displayed significant and comparable antitumor effects compared with the vehicle control group, while Anlotinib at 2 mg/kg did not inhibit tumor growth (**Supplementary Figure S1A**), suggesting that the effective low-dose of Anlotinib for LAP0297 lung tumor model is 4 mg/kg. We then treated LAP0297 tumor-bearing mice with Anlotinib (4 mg/kg), anti-PD-1 therapy, or their combination. Either Anlotinib (4 mg/kg) or anti-PD-1 therapy slightly retarded LAP0297 lung tumor growth, while the combination therapy dramatically inhibited tumor growth compared with all the other groups (**Supplementary Figure S1B**). The data show that effective low-dose Anlotinib treatments synergistically improve the efficacy of anti-PD-1 therapy in the LAP0297 lung tumor model.

**Figure 3:**
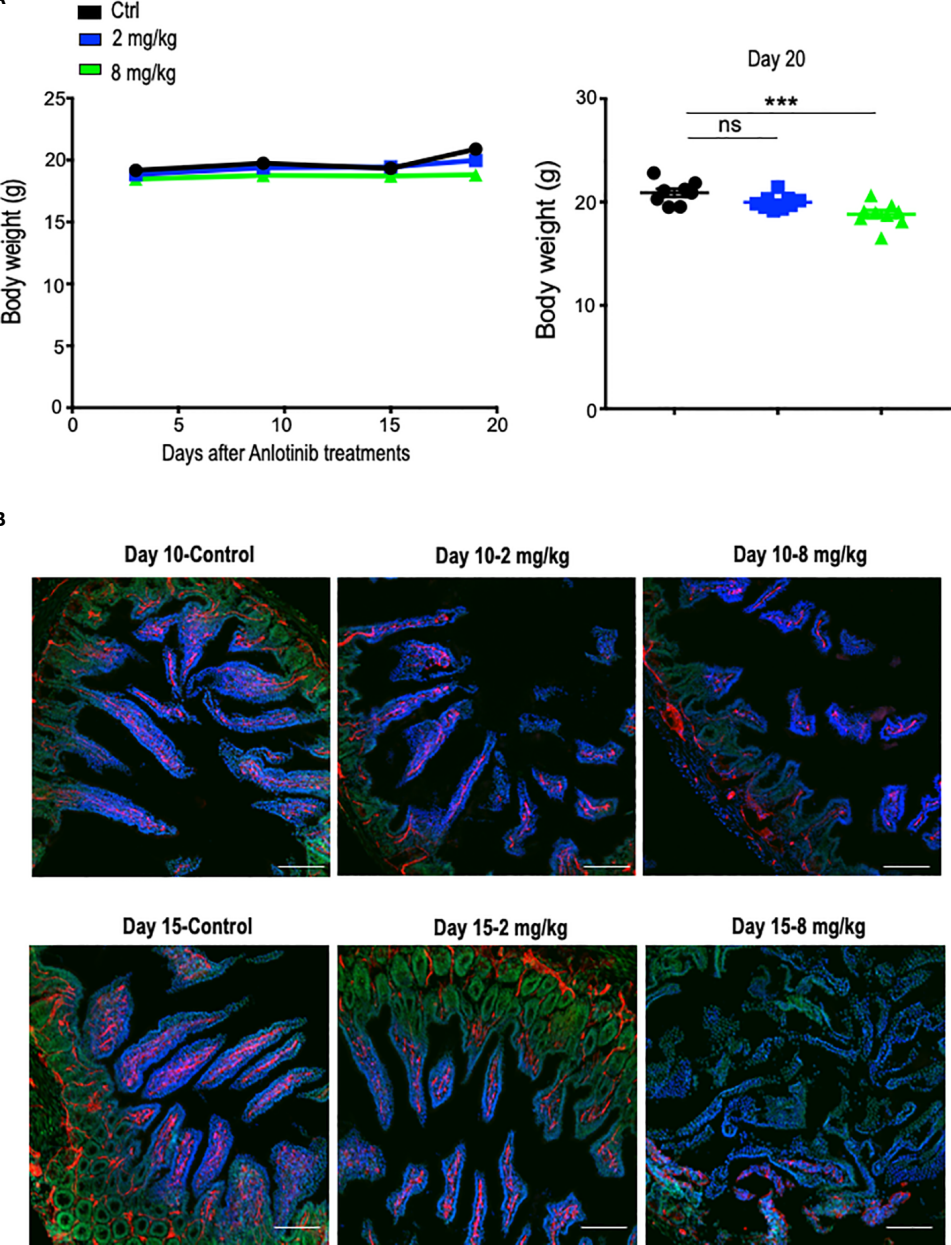
Long-term effective low-dose Anlotinib treatments exhibited fewer side effects compared to high-dose. EO771 tumor-bearing mice were prepared, and Anlotinib treatments were conducted as described in **Figure 2**. The duration of Anlotinib treatments was 20 days. Prior to colon tissue collection, mice were intravenously injected with 200 µg/mouse Hoechst 33342 (Ho33342). **(A)** The body weight of mice was monitored every 5 days post-Anlotinib treatments. The significance was determined by one-way ANOVA (*n* = 8–9 mice per group). The data are presented as means ± SEM. ns, no significant difference; ^***^ *p* < 0.001. **(B)** Representative images of colon tissues from vehicle control and mice treated with low- or high-dose Anlotinib. Each group had eight to nine mice. Ho33342 perfusion (blue) indicating blood vessel function and CD31 (red) staining blood vessels. Scale bar=100 µm.

The combination of immune checkpoint therapy and antiangiogenic treatments is commonly administered in a long-term regimen in the clinic. Thus, we conducted a long-term combination of Anlotinib and anti-PD-1 therapy in the orthotopic EO771 breast tumor model. Long-term low-dose Anlotinib (2 mg/kg) treatments combined with anti-PD-1 therapy were more effective in inhibiting tumor growth compared with both the monotherapy (**Figure 2B**). By contrast, long-term high-dose Anlotinib (8 mg/kg) plus anti-PD-1 therapy did not induce more tumor growth inhibition as compared to high-dose Anlotinib monotherapy (**Figure 2B**). However, body weight loss was detected in mice treated with long-term high-dose, but not low-dose, Anlotinib (8 mg/kg), compared with the vehicle control group (**Figure 3A**). Moreover, after 10 days of high-dose Anlotinib (8 mg/kg) treatments, the number of small intestinal villi decreased and the structure was damaged compared with those of vehicle control or long-term low-dose group, which were more severe after 15 days of high-dose Anlotinib treatments (**Figure 3B**). Together, these results show that effective low-dose Anlotinib treatments improve anti-PD-1 therapy in both short- and long-term regimens with reduced side effects in the EO771 breast tumor model.

### Effective low-dose Anlotinib treatments induce long-term tumor vascular normalization and its combination with anti-PD-1 therapy increases the percentages of intratumoral CD4^+^ T, CD8^+^ T, and NK cells

**Figure 4:**
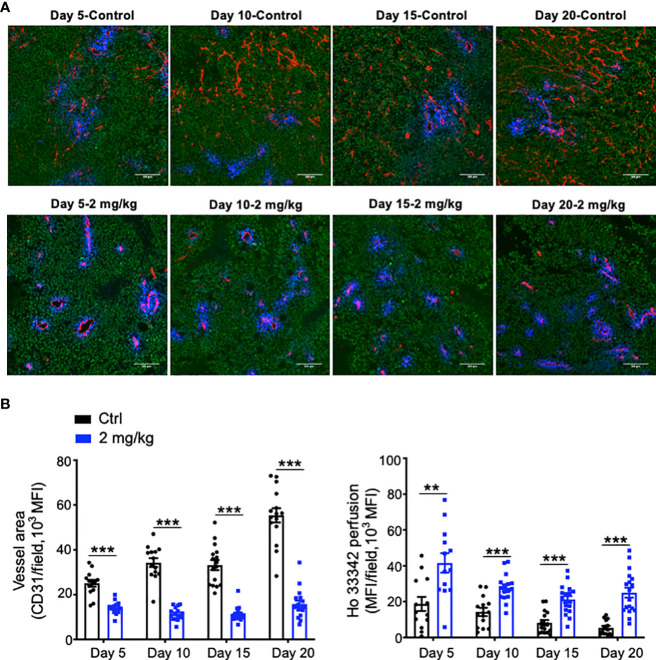
Effective low-dose Anlotinib treatments induced long-term tumor vascular normalization. EO771 breast tumor-bearing mice were prepared and Anlotinib treatments (2 mg/kg/daily) were conducted as described in **Figure 2**. Tumor tissues were harvested on Days 5, 10, 15, and 20 after vehicle control or Anlotinib treatments. **(A)** Representative images of Ho33342 perfusion (blue) and CD31-staining (red). Scale bar=100 µm. **(B)** Ho33342 perfused tumor areas and vessel density (CD31). The significance was determined by unpaired Student’s *t*-test (n=13-19 tumors per group). The data are presented as means ± SEM. **P < 0.01; ***P < 0.001.

**Figure 5:**
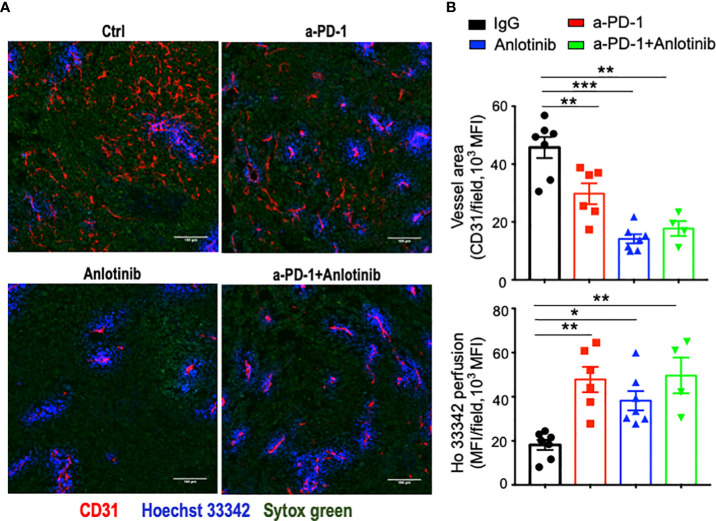
Either anti-PD-1 therapy or its combination with effective low-dose Anlotinib induced tumor vascular normalization. EO771 breast tumor-bearing mice were prepared, Anlotinib treatments (2 mg/kg/daily), and anti-PD-1 therapy (5 mg/kg, every three days) were conducted as described in **Figure 2**. Treatment duration was one week. **(A)** Representative images of Ho33342 perfusion (blue) and CD31-staining (red). Scale bar = 100 µm. **(B)** Ho33342 perfused tumor areas and vessel density (CD31). The significance was determined by one-way ANOVA (*n* = 4–7 mice per group). The data are presented as means ± SEM. ^*^ *p* < 0.05; ^**^ *p* < 0.01; ^***^ *p* < 0.001.

**Figure 6:**
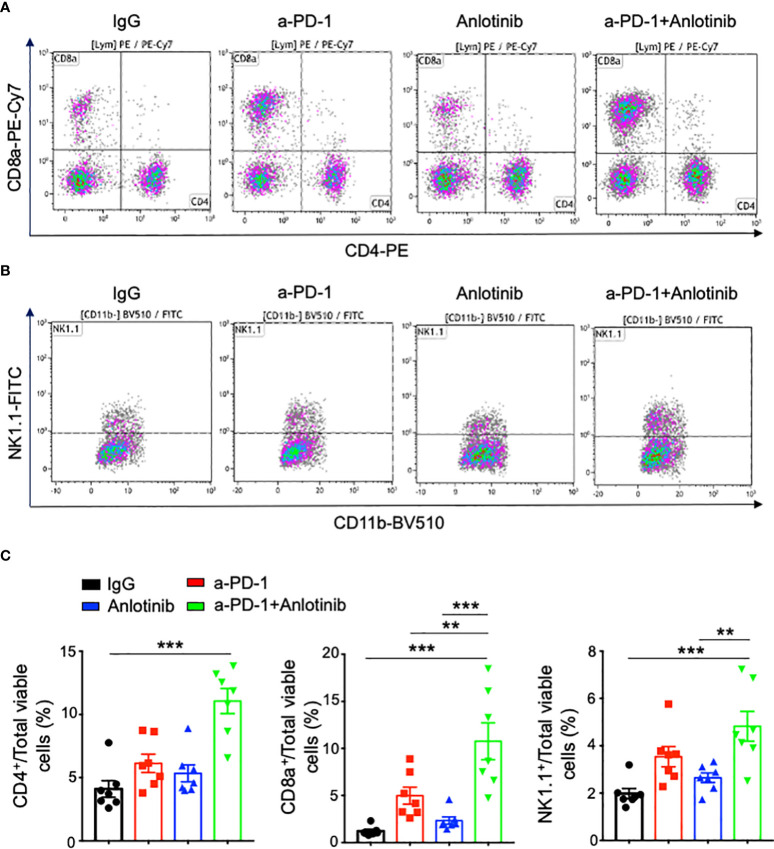
The combination of effective low-dose Anlotinib and anti-PD-1 therapy increased the percentages of intratumoral T and NK cells. EO771 breast tumor-bearing mice and the combination of Anlotinib and anti-PD-1 therapy were conducted as described in **Figure 4**. Tumor tissues were collected and analyzed by flow cytometric analysis. **(A, B)** Representative flow cytometric plots of intratumoral CD4^+^, CD8^+^ T, and NK cells. **(C)** The percentages of intratumoral CD4^+^, CD8^+^ T, and NK cells. The significance was determined by one-way ANOVA (*n* = 7 mice per group). The data are presented as means ± SEM. ^**^ *p* < 0.01; ^***^ *p* < 0.001.

Abnormal tumor vessels and their resulting immunosuppressive tumor microenvironment are major challenges facing immune checkpoint blockade therapy (7, 31). Either low-dose antiangiogenic therapy or immune checkpoint blockade therapy can induce tumor vascular normalization and recondition of the tumor immune microenvironment (31, 32, 37, 38). However, the duration of vascular normalization upon low-dose antiangiogenic therapy is unknown. Thus, we harvested tumor tissues from days 5 to 20 after effective low-dose Anlotinib treatments. Indeed, effective low-dose Anlotinib treatments reduced tumor blood vessel density while elevating vascular perfusion compared with the control group on day 5, suggesting the induction of tumor vascular normalization (**Figure 4**). Remarkably, the reduction of tumor vessel density and the improvement of vessel perfusion were also significant from days 10 to 20 (**Figure 4**). These data show that effective low-dose Anlotinib induces long-term tumor vascular normalization. We further analyzed the impacts of Anlotinib and anti-PD-1 combination therapy on the tumor vasculature. Anti-PD-1 therapy or its combination with effective low-dose Anlotinib treatments significantly reduced tumor blood vessel density while elevating vascular perfusion compared with the isotype control group, suggesting the induction of tumor vascular normalization (**Figure 5**). We then analyzed the impacts of those treatments on the tumor immune microenvironment. The percentages of intratumoral CD4^+^ T, CD8^+^ T, and NK cells were significantly increased in the combination group compared with the isotype control and monotherapy groups (**Figure 6**). The proportions of immunosuppressive CD4^+^CD25^+^ T cells were comparable in all groups (**Supplementary Figures S2**, **S3**). The proportions of PD1^+^CD4^+^/CD4^+^ T cells were decreased, while the proportions of PD1^+^CD8^+^/CD8^+^ T cells were increased in the anti-PD-1 or combination treatment groups compared with the isotype control group (**Supplementary Figure S3**). We also analyzed myeloid cell populations in tumor tissues. Effective low-dose Anlotinib treatments, anti-PD-1 therapy, or their combination therapy did not change the proportions of intratumoral M-MDSCs (CD45^+^CD11b^+^Gr1^med^F4/80^med^) and PMN-MDSCs (CD45^+^CD11b^+^Gr1^hi^F4/80^−^) (**Supplementary Figure S4**). Together, these results show that effective low-dose Anlotinib combined with anti-PD-1 therapy induces tumor vascular normalization and increases the proportions of CD4^+^ T, CD8^+^ T, and NK cells in EO771 breast tumors, which may, in turn, contribute to enhanced antitumor effects.

### CD8^+^ T cells partially mediate the antitumor effects of the combination therapy, while Anlotinib treatments inhibit tumor growth independent of IFN-γ

**Figure 7:**
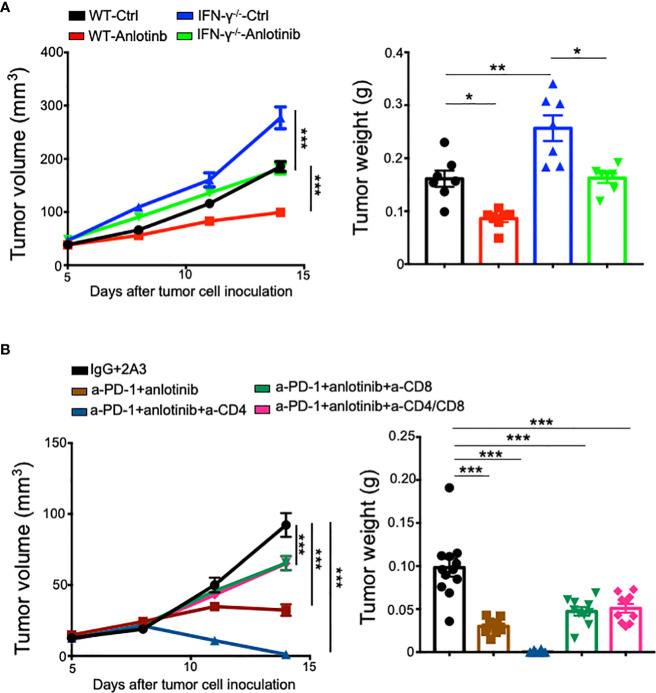
*In vivo* depletion of CD8^+^ T cells partially reversed the antitumor effects of the combination therapy, while Anlotinib treatments inhibited tumor growth independent of IFN-γ. WT or IFN-γ^−/−^ mice were orthotopically inoculated with 2 × 10^5^ EO771 breast tumor cells. When EO771 breast tumors reached 3–4 mm in diameter, mice were randomly divided into appropriate groups and treated with ddH_2_O or Anlotinib (2 mg/kg/daily), IgG, or anti-PD-1 antibodies (5 mg/kg every 3 days). Some groups of mice were also treated with isotype 2A3, anti-CD8, or/and anti-CD4 antibodies (200 µg/mouse) on days 5, 7, and 12 after tumor cell inoculation. **(A)** The impacts of IFN-γ on tumor growth upon Anlotinib treatments. **(B)** The influences of T-cell depletion on tumor growth upon Anlotinib and anti-PD-1 combination therapy. The significance was determined by one-way ANOVA (*n* = 7 mice per group in A, and *n* = 10–12 mice per group in **B**). The data are presented as means ± SEM. ^*^ *p* < 0.05; ^**^ *p* < 0.01; ^***^ *p* < 0.001.

It is well-known that CD4^+^ and CD8^+^ T cells, as well as IFN-γ are critical for antitumor immunity (31, 39, 40). To investigate the role of IFN-γ in Anlotinib treatments, we used an *Ifnγ* knockout mouse model. EO771 tumors grew faster in *Ifnγ* knockout mice compared with wild-type control mice. Meanwhile, Anlotinib treatments still suppressed tumor growth effectively in *Ifnγ* knockout mice compared with the vehicle control group (**Figure 7A**). The data suggest that Anlotinib inhibits tumor growth independent of IFN-γ. As the combination therapy increased the proportions of intratumoral CD4^+^ and CD8^+^ T cells, we next investigated whether T cells mediate the antitumor effects of the combination therapy. *In vivo* depletion of CD4^+^ and CD8^+^ T cells simultaneously or CD8^+^ T cells alone partially reversed tumor growth inhibition induced by the combination of effective low-dose Anlotinib and anti-PD-1 therapy in EO771 tumors. Unexpectedly, *in vivo* depletion of CD4^+^ T cells alone resulted in complete regression of EO771 tumors, while the combination of Anlotinib and anti-PD-1 therapy only slowed down EO771 tumor growth (**Figure 7B**). In summary, these results show that the antitumor effects of the combination of effective low-dose Anlotinib and anti-PD-1 therapy are partially mediated by CD8^+^ T cells while Anlotinib treatments inhibit tumor growth independent of IFN-γ.

## Discussion

Immune checkpoint blockade therapy has achieved long-term efficacy in a small fraction of cancer patients. Its combination with antiangiogenic treatments, including bevacizumab, axitinib, and lenvatinib, showed promising clinical benefits (34–36, 41). The process of optimizing the combination of antiangiogenic agents and immune checkpoint inhibitors to achieve better efficacy while reducing adverse influences remains not fully understood. In this study, we found that a relatively low-dose of Anlotinib was sufficient to achieve maximum antitumor effects with fewer side effects compared with a high-dose. Notably, effective low-dose Anlotinib induced long-lasting tumor vascular normalization. The combination of effective low-dose Anlotinib and anti-PD-1 therapy increased the proportions of intratumoral CD4^+^ T, CD8^+^ T, and NK cells. Moreover, effective low-dose Anlotinib treatments enhanced anti-PD-1 therapy in both short- and long-term treatment regimens, partially depending on CD8^+^ T cells. Our findings suggest that effective low-dose Anlotinib treatments induced durable tumor vascular normalization and persistently enhanced anti-PD-1 therapy, providing preclinical evidence to develop more effective and safer combinational anti-PD-1therapy by using effective low-dose Anlotinib.

The antitumor effects of an antiangiogenic agent are proposed to attributed to tumor blood vessel pruning, limiting the supply of nutrients to tumor cells (6, 8). Higher dosages of antiangiogenic agents often exhibit better vessel pruning and antitumor effects. Interestingly, relatively low-dose Anlotinib treatments exhibited similar tumor growth inhibition, compared with those of medium and high dosages. Moreover, relatively low-dose Anlotinib treatments dramatically reduced tumor blood vessel density, and increased Anlotinib dosage did not enhance vessel pruning compared with a relatively low-dose. These results indicate that Anlotinib is a very potent antiangiogenic agent; therefore, a relatively low-dose Anlotinib is sufficient to realize its antitumor and antiangiogenic effects.

Previous studies have suggested that low-dose antiangiogenic therapy induces tumor vascular normalization, reduces the compactness of tumors to relieve solid stress, increases the infiltration of immune effector cells, and converts the immunosuppressive tumor microenvironment to improve the efficacy of cancer immunotherapy (8, 32, 38, 42, 43). Here, we further showed that relatively low-dose Anlotinib treatments induced long-lasting tumor vascular normalization and potentiated anti-PD-1 therapy in both short- and long-term treatment regimens. In comparison, relatively high-dose Anlotinib treatments only improved anti-PD-1 therapy in the short-term treatment regimen. This is presumably due to the durable tumor vascular normalization induced by relatively low-dose Anlotinib treatments, while the duration of tumor vascular normalization induced by relatively high-dose Anlotinib treatments is likely transient. A recent report showed that Anlotinib treatments can induce tumor vascular normalization, which disappeared following its long-term treatments (27). Based on these results, we proposed the “effective low-dose” concept for Anlotinib therapy and potentially for other antiangiogenic therapies. An effective low-dose would be an optimal dosage for Anlotinib to significantly inhibit tumor growth and induce tumor vascular normalization while avoiding intolerable side effects.

The formation of a positive feedback loop between tumor vascular normalization and immune cell activation could be a novel mechanism for durable antitumor effects (31, 44). The combination of low-dose Anlotinib and anti-PD-1 therapy elevated the percentages of intratumoral CD4^+^ T, CD8^+^ T, and NK cells, and its antitumor effects were partially dependent on CD8^+^ T cells, indicating the activation of CD8^+^ T cells by the combination therapy. A recent study suggested that Anlotinib treatments downregulated PD-L1 expression in tumor endothelial cells to alleviate the immunosuppressive barrier and increase CD8^+^ T-cell tumor infiltration (45). Consistently, we observed that long-term Anlotinib treatments at the dose of 2 mg/kg, but not 8 mg/kg, reduced the levels of PD-L1 expression in tumor endothelial cells compared with control tumor blood vessels (**Supplementary Figure S5**), which may contribute to the increased tumor infiltration of CD8^+^ T cells upon Anlotinib treatments. Activated CD8^+^ T cells may also promote tumor vascular normalization. Hence, it is likely that Anlotinib and activated CD8^+^ T cells coordinated together to induce tumor vascular normalization. This may explain why low-dose, but not high-dose, Anlotinib treatments induced tumor vascular normalization and improved anti-PD-1 therapy in the long-term treatment regimen.

It is well-known that CD4^+^ and CD8^+^ T cells play crucial roles in antitumor immune responses (46–49). *In vivo* depletion of CD8^+^ T cells partially reversed the antitumor effects of the combination of low-dose Anlotinib and anti-PD-1 therapy. In addition, Anlotinib treatments inhibited tumor growth independent of IFN-γ. Together, these results indicate that Anlotinib could exert tumor growth inhibition *via* non-T-cell mechanisms such as tumor blood vessel pruning and direct tumor cell cytotoxicity. Another possibility is that NK cells may involve in the antitumor effects of Anlotinib because Anlotinib monotherapy or its combination with anti-PD-1 antibody increased the percentages of intratumoral NK cells. Interestingly, *in vivo* depletion of CD4^+^ T cells potentiated the combination of low-dose Anlotinib and anti-PD-1 therapy, leading to tumor regression. This could be due to the deletion of immunosuppressive CD4^+^CD25^+^ T cells, which relieves the suppressive effects on antitumor immunity induced by anti-PD-1 therapy.

In summary, this study provides preclinical evidence that relatively low-dose Anlotinib treatments are sufficient to achieve optimal antitumor effects. Low-dose Anlotinib treatments normalize tumor blood vessels, reprogram the tumor immune microenvironment, and enhance anti-PD-1 therapy in both short- and long-term treatment regimens, while minimizing side effects. Thus, we proposed that Anlotinib, in particular, and antiangiogenic agents, in general, should be treated at an effective low-dose, rather than a tolerable high-dose, because it would be more efficacious, safe, and economic.

## Data availability statement

The original contributions presented in the study are included in the article/**Supplementary Material**. Further inquiries can be directed to the corresponding authors.

## Ethics statement

The animal study was reviewed and approved by The Institutional Laboratory Animal Care and Use Committee of Soochow University.

## Author contributions

PF, HQ, ZL, YW, and TL performed the experiments. PF, XW, SQ, WX, and YH analyzed the data, prepared the figures, and interpreted the results. QZ and TC provided scientific inputs and technical support. PF wrote the draft of the manuscript. YH, WX, and SQ designed the experiments, supervised the research, and wrote the manuscript with inputs from all of the other authors. All authors contributed to the article and approved the submitted version.

## Funding

This work was supported in part by grants from the National Natural Science Foundation of China (82073337 to SQ and 81972877 to YH), Jiangsu Key Laboratory for Carbon-Based Functional Materials & Devices, Soochow University, the Collaborative Innovation Center of Hematology, and the Priority Academic Program Development of Jiangsu Higher Education Institutions.

## Acknowledgments

We thank Naidong Zhang and Long Qian for their technical support.

## Conflict of interest

Authors XW and WX were employed by Innovent Biologics Inc. (Suzhou, China).

The remaining authors declare that the research was conducted in the absence of any commercial or financial relationships that could be construed as a potential conflict of interest.

## Publisher’s note

All claims expressed in this article are solely those of the authors and do not necessarily represent those of their affiliated organizations, or those of the publisher, the editors and the reviewers. Any product that may be evaluated in this article, or claim that may be made by its manufacturer, is not guaranteed or endorsed by the publisher.

## References

[1] Folkman J. Tumor angiogenesis: therapeutic implications. N Engl J Med (1971) 285(21):1182–6. doi: 10.1056/NEJM197111182852108 4938153

[2] Carmeliet P Jain RK. Molecular mechanisms and clinical applications of angiogenesis. Nature (2011) 473(7347):298–307. doi: 10.1038/nature10144 21593862 PMC4049445

[3] Bergers G Hanahan D. Modes of resistance to anti-angiogenic therapy. Nat Rev Cancer (2008) 8(8):592–603. doi: 10.1038/nrc2442 18650835 PMC2874834

[4] Gacche RN Assaraf YG. Redundant angiogenic signaling and tumor drug resistance. Drug Resist Update (2018) 36:47–76. doi: 10.1016/j.drup.2018.01.002 29499837

[5] Qin S Li A Yi M Yu S Zhang M Wu K. Recent advances on anti-angiogenesis receptor tyrosine kinase inhibitors in cancer therapy. J Hematol Oncol (2019) 12(1):27. doi: 10.1186/s13045-019-0718-5 30866992 PMC6417086

[6] Eelen G Treps L Li X Carmeliet P. Basic and therapeutic aspects of angiogenesis updated. Circ Res (2020) 127(2):310–29. doi: 10.1161/CIRCRESAHA.120.316851 32833569

[7] Matuszewska K Pereira M Petrik D Lawler J Petrik J. Normalizing tumor vasculature to reduce hypoxia, enhance perfusion, and optimize therapy uptake. Cancers (Basel) (2021) 13(17):4444–63. doi: 10.3390/cancers13174444 PMC8431369 34503254

[8] Fukumura D Kloepper J Amoozgar Z Duda DG Jain RK. Enhancing cancer immunotherapy using antiangiogenics: opportunities and challenges. Nat Rev Clin Oncol (2018) 15(5):325–40. doi: 10.1038/nrclinonc.2018.29 PMC5921900 29508855

[9] Jain RK. Antiangiogenesis strategies revisited: from starving tumors to alleviating hypoxia. Cancer Cell (2014) 26(5):605–22. doi: 10.1016/j.ccell.2014.10.006 PMC4269830 25517747

[10] Wilson WR Hay MP. Targeting hypoxia in cancer therapy. Nat Rev Cancer (2011) 11(6):393–410. doi: 10.1038/nrc3064 21606941

[11] Batchelor TT Sorensen AG di Tomaso E Zhang WT Duda DG Cohen KS. AZD2171, a pan-VEGF receptor tyrosine kinase inhibitor, normalizes tumor vasculature and alleviates edema in glioblastoma patients. Cancer Cell (2007) 11(1):83–95. doi: 10.1016/j.ccr.2006.11.021 17222792 PMC2748664

[12] Sorensen AG Emblem KE Polaskova P Jennings D Kim H Ancukiewicz M. Increased survival of glioblastoma patients who respond to antiangiogenic therapy with elevated blood perfusion. Cancer Res (2012) 72(2):402–7. doi: 10.1158/0008-5472.CAN-11-2464 PMC3261301 22127927

[13] Sorensen AG Batchelor TT Zhang WT Chen PJ Yeo P Wang M. A "vascular normalization index" as potential mechanistic biomarker to predict survival after a single dose of cediranib in recurrent glioblastoma patients. Cancer Res (2009) 69(13):5296–300. doi: 10.1158/0008-5472.CAN-09-0814 PMC2824172 19549889

[14] Thews O Riemann A Nowak M Gekle M. Impact of hypoxia-related tumor acidosis on cytotoxicity of different chemotherapeutic drugs *in vitro* and in vivo. Adv Exp Med Biol (2014) 812:51–8. doi: 10.1007/978-1-4939-0620-8_7 24729214

[15] Sanna K Rofstad EK. Hypoxia-induced resistance to doxorubicin and methotrexate in human melanoma cell lines in vitro. Int J Cancer (1994) 58(2):258–62. doi: 10.1002/ijc.2910580219 8026888

[16] Graham K Unger E. Overcoming tumor hypoxia as a barrier to radiotherapy, chemotherapy and immunotherapy in cancer treatment. Int J Nanomed (2018) 13:6049–58. doi: 10.2147/IJN.S140462 PMC6177375 30323592

[17] Jain RK. Normalization of tumor vasculature: an emerging concept in antiangiogenic therapy. Science (2005) 307(5706):58–62. doi: 10.1126/science.1104819 15637262

[18] Jain RK. Normalizing tumor microenvironment to treat cancer: bench to bedside to biomarkers. J Clin Oncol (2013) 31(17):2205–18. doi: 10.1200/JCO.2012.46.3653 PMC3731977 23669226

[19] Matsumoto S Saito K Takakusagi Y Matsuo M Munasinghe JP Morris HD. *In vivo* imaging of tumor physiological, metabolic, and redox changes in response to the anti-angiogenic agent sunitinib: longitudinal assessment to identify transient vascular renormalization. Antioxid Redox Signal (2014) 21(8):1145–55. doi: 10.1089/ars.2013.5725 PMC4142786 24597714

[20] Huang Y Stylianopoulos T Duda DG Fukumura D Jain RK. Benefits of vascular normalization are dose and time dependent–letter. Cancer Res (2013) 73(23):7144–6. doi: 10.1158/0008-5472.CAN-13-1989 PMC3876035 24265277

[21] Ebos JM Kerbel RS. Antiangiogenic therapy: impact on invasion, disease progression, and metastasis. Nat Rev Clin Oncol (2011) 8(4):210–21. doi: 10.1038/nrclinonc.2011.21 PMC4540336 21364524

[22] Jayson GC Kerbel R Ellis LM Harris AL. Antiangiogenic therapy in oncology: current status and future directions. Lancet (2016) 388(10043):518–29. doi: 10.1016/S0140-6736(15)01088-0 26853587

[23] Han B Li K Wang Q Zhang L Shi J Wang Z. Effect of anlotinib as a third-line or further treatment on overall survival of patients with advanced non-small cell lung cancer: The ALTER 0303 phase 3 randomized clinical trial. JAMA Oncol (2018) 4(11):1569–75. doi: 10.1001/jamaoncol.2018.3039 PMC6248083 30098152

[24] Chen XZ. Anlotinib for refractory advanced non-small cell lung cancer in China. JAMA Oncol (2019) 5(1):116–7. doi: 10.1001/jamaoncol.2018.5526 30489609

[25] Zhang X Zeng L Li Y Xu Q Yang H Lizaso A. Anlotinib combined with PD-1 blockade for the treatment of lung cancer: a real-world retrospective study in China. Cancer Immunol Immunother (2021) 70(9):2517–28. doi: 10.1007/s00262-021-02869-9 PMC10991983 33566148

[26] Yang Y Li L Jiang Z Wang B Pan Z. Anlotinib optimizes anti-tumor innate immunity to potentiate the therapeutic effect of PD-1 blockade in lung cancer. Cancer Immunol Immunother (2020) 69(12):2523–32. doi: 10.1007/s00262-020-02641-5 PMC11027453 32577817

[27] Su Y Luo B Lu Y Wang D Yan J Zheng J. Anlotinib induces a T cell-inflamed tumor microenvironment by facilitating vessel normalization and enhances the efficacy of PD-1 checkpoint blockade in neuroblastoma. Clin Cancer Res (2022) 28(4):793–809. doi: 10.1158/1078-0432.CCR-21-2241 34844980 PMC9377760

[28] Zhou N Jiang M Li T Zhu J Liu K Hou H. Anlotinib combined with anti-PD-1 antibody, camrelizumab for advanced NSCLCs after multiple lines treatment: An open-label, dose escalation and expansion study. Lung Cancer (2021) 160:111–7. doi: 10.1016/j.lungcan.2021.08.006 34482102

[29] Xu W Wang K Gu W Nie X Zhang H Tang C. Case report: Complete remission with anti-PD-1 and anti-VEGF combined therapy of a patient with metastatic primary splenic angiosarcoma. Front Oncol (2022) 12:809068. doi: 10.3389/fonc.2022.809068 35311098 PMC8928100

[30] Yuan M Zhu Z Mao W Wang H Qian H Wu J. Anlotinib combined with anti-PD-1 antibodies therapy in patients with advanced refractory solid tumors: A single-center, observational, prospective study. Front Oncol (2021) 11:683502. doi: 10.3389/fonc.2021.683502 34692475 PMC8529018

[31] Zheng X Fang Z Liu X Deng S Zhou P Wang X. Increased vessel perfusion predicts the efficacy of immune checkpoint blockade. J Clin Invest (2018) 128(5):2104–15. doi: 10.1172/JCI96582 PMC5957454 29664018

[32] Huang Y Yuan J Righi E Kamoun WS Ancukiewicz M Nezivar J. Vascular normalizing doses of antiangiogenic treatment reprogram the immunosuppressive tumor microenvironment and enhance immunotherapy. Proc Natl Acad Sci U.S.A. (2012) 109(43):17561–6. doi: 10.1073/pnas.1215397109 PMC3491458 23045683

[33] Huinen ZR Huijbers EJM van Beijnum JR Nowak-Sliwinska P Griffioen AW. Anti-angiogenic agents - overcoming tumour endothelial cell anergy and improving immunotherapy outcomes. Nat Rev Clin Oncol (2021) 18(8):527–40. doi: 10.1038/s41571-021-00496-y 33833434

[34] Socinski MA Jotte RM Cappuzzo F Orlandi F Stroyakovskiy D Nogami N. Atezolizumab for first-line treatment of metastatic nonsquamous NSCLC. N Engl J Med (2018) 378(24):2288–301. doi: 10.1056/NEJMoa1716948 29863955

[35] Ren Z Xu J Bai Y Xu A Cang S Du C. Sintilimab plus a bevacizumab biosimilar (IBI305) versus sorafenib in unresectable hepatocellular carcinoma (ORIENT-32): a randomised, open-label, phase 2-3 study. Lancet Oncol (2021) 22(7):977–90. doi: 10.1016/S1470-2045(21)00252-7 34143971

[36] Finn RS Qin S Ikeda M Galle PR Ducreux M Kim TY. Atezolizumab plus bevacizumab in unresectable hepatocellular carcinoma. N Engl J Med (2020) 382(20):1894–905. doi: 10.1056/NEJMoa1915745 32402160

[37] Tian L Goldstein A Wang H Ching Lo H Sun Kim I Welte T. Mutual regulation of tumour vessel normalization and immunostimulatory reprogramming. Nature (2017) 544(7649):250–4. doi: 10.1038/nature21724 PMC5788037 28371798

[38] Zhao S Ren S Jiang T Zhu B Li X Zhao C. Low-dose apatinib optimizes tumor microenvironment and potentiates antitumor effect of PD-1/PD-L1 blockade in lung cancer. Cancer Immunol Res (2019) 7(4):630–43. doi: 10.1158/2326-6066.CIR-17-0640 30755403

[39] Shankaran V Ikeda H Bruce AT White JM Swanson PE Old LJ. IFNgamma and lymphocytes prevent primary tumour development and shape tumour immunogenicity. Nature (2001) 410(6832):1107–11. doi: 10.1038/35074122 11323675

[40] Zhang M Huang L Ding G Huang H Cao G Sun X. Interferon gamma inhibits CXCL8-CXCR2 axis mediated tumor-associated macrophages tumor trafficking and enhances anti-PD1 efficacy in pancreatic cancer. J Immunother Cancer (2020) 8(1):1–15. doi: 10.1136/jitc-2019-000308 PMC7057481 32051287

[41] Makker V Colombo N Casado Herraez A Santin AD Colomba E Miller DS. Lenvatinib plus pembrolizumab for advanced endometrial cancer. N Engl J Med (2022) 386(5):437–48. doi: 10.1056/NEJMoa2108330 PMC11651366 35045221

[42] Johansson A Hamzah J Payne CJ Ganss R. Tumor-targeted TNFalpha stabilizes tumor vessels and enhances active immunotherapy. Proc Natl Acad Sci U.S.A. (2012) 109(20):7841–6. doi: 10.1073/pnas.1118296109 PMC3356673 22547817

[43] Li Q Wang Y Jia W Deng H Li G Deng W. Low-dose anti-angiogenic therapy sensitizes breast cancer to PD-1 blockade. Clin Cancer Res (2020) 26(7):1712–24. doi: 10.1158/1078-0432.CCR-19-2179 31848190

[44] Huang Y Kim BYS Chan CK Hahn SM Weissman IL Jiang W. Improving immune-vascular crosstalk for cancer immunotherapy. Nat Rev Immunol (2018) 18(3):195–203. doi: 10.1038/nri.2017.145 29332937 PMC5922422

[45] Liu S Qin T Liu Z Wang J Jia Y Feng Y. Anlotinib alters tumor immune microenvironment by downregulating PD-L1 expression on vascular endothelial cells. Cell Death Dis (2020) 11(5):309. doi: 10.1038/s41419-020-2511-3 32366856 PMC7198575

[46] Carretero R Sektioglu IM Garbi N Salgado OC Beckhove P Hammerling GJ. Eosinophils orchestrate cancer rejection by normalizing tumor vessels and enhancing infiltration of CD8(+) T cells. Nat Immunol (2015) 16(6):609–17. doi: 10.1038/ni.3159 25915731

[47] Jin YW Hu P. Tumor-infiltrating CD8 T cells predict clinical breast cancer outcomes in young women. Cancers (Basel) (2020) 12(5):1076–90. doi: 10.3390/cancers12051076 PMC7281139 32357420

[48] Mahmoud SM Paish EC Powe DG Macmillan RD Grainge MJ Lee AH. Tumor-infiltrating CD8+ lymphocytes predict clinical outcome in breast cancer. J Clin Oncol (2011) 29(15):1949–55. doi: 10.1200/JCO.2010.30.5037 21483002

[49] Zhang N Yin R Zhou P Liu X Fan P Qian L. DLL1 orchestrates CD8(+) T cells to induce long-term vascular normalization and tumor regression. Proc Natl Acad Sci U.S.A. (2021) 118(22):1–9. doi: 10.1073/pnas.2020057118 PMC8179177 34035167

