# Supplementary material for: Effective low-dose Anlotinib induces long-term tumor vascular normalization and improves anti-PD-1 therapy
Source: Front Immunol. 2022 Aug 3;13:937924. doi: 10.3389/fimmu.2022.937924 (PMC9382125; doi:10.3389/fimmu.2022.937924)

Supplementary figure legends

Figure. S1 | Low dose Anlotinib treatments synergistically improved anti-PD-1 therapy in LAP0297 lung tumors. Female FVB mice were subcutaneously injected with 5×10^4 LAP0297 lung tumor cells. When LAP0297 lung tumors reached 3×4 mm in diameter, mice were randomly grouped for different treatments. Tumor volume was monitored every 3 days and the tumor weight was measured at the end of the experiments. (**A**) Tumor growth curves and the weight of LAP0297 lung tumors after different doses of Anlotinib treatments. (**B**) Tumor growth curves and the weight of LAP0297 lung tumors after Anlotinib, anti-PD-1, or their combination therapy. When tumors reached 3×4 mm in diameter, mice were randomly grouped and administered with ddH_2O or Anlotinib (4 mg/kg) daily by oral gavage, or anti–PD-1 as well as isotype IgG (2.5 mg/kg) via the tail vein every 3 days for 5 times. The significance was determined by one-way ANOVA (n=5 mice per group in A, and n=8 mice per group in B). The data are presented as means ± SEM. *$P < 0.05$, **$P < 0.01$, ***$P < 0.001$.

FIGURE S2 | Gating methods of multicolor flow cytometric analysis to identify the subtypes of tumor-infiltrating immune cell populations.

FIGURE S3 | The impacts of Anlotinib and anti-PD-1 treatments on T cells in EO771 breast tumors. EO771 tumors were prepared and treated as described in Fig. 4 and 5. (**A-C**) The representative flow cytometric profiles of intra-tumoral Tregs, $PD1^+CD4^+$ and $PD1^+CD8^+$ T cells. (**D-F**) The proportions of intra-tumoral Tregs, $PD1^+CD4^+$ and $PD1^+CD8^+$ T cells. The significance was determined by one-way ANOVA (n=7 mice per group). The data are presented as means ± SEM. *$P < 0.05$, **$P < 0.01$, ***$P < 0.001$.

FIGURE S4 | The effects of the combination of Anlotinib and anti-PD-1 treatments on myeloid cells in EO771 breast tumors. EO771 tumors were prepared and treated as described in Fig. 4 and 5. (**A, B**) The representative flow cytometric profiles of intra-

tumoral TAMs, monocytes and neutrophils. (**C-H**) The proportions of intra-tumoral TAMs, M-MDSCs, PMN-MDSCs as well as M1- and M2-like TAMs. The significance was determined by one-way ANOVA (n=7 mice per group). The data are presented as means ± SEM. **P* < 0.05, ****P* < 0.001.

FIGURE S5| Low dose Anlotinib treatments reduced PD-L1 expression in tumor endothelial cells. EO771 tumor-bearing mice were prepared and Anlotinib treatments (2 or 8 mg/kg/daily) were conducted as described in Fig. 2B. Tumor tissues were harvested on Day 20 after vehicle control or Anlotinib treatments. Five minutes prior to tumor tissue harvest, mice were intravenously injected with 200 μg/mouse Hoechst 33342 (Ho33342). The tissue sections were stained with anti-PD-L1 and anti-CD31. Representative images were showed. Scale bar=50 μm. Ho33342 (blue), Hoechst 33342 perfused area; CD31 (red), endothelial cells; PD-L1 (green), expression of PD-L1 in tumor tissue cells; yellow arrows showing the colocalization of PD-L1 and CD31 stainings.

Fig. S1

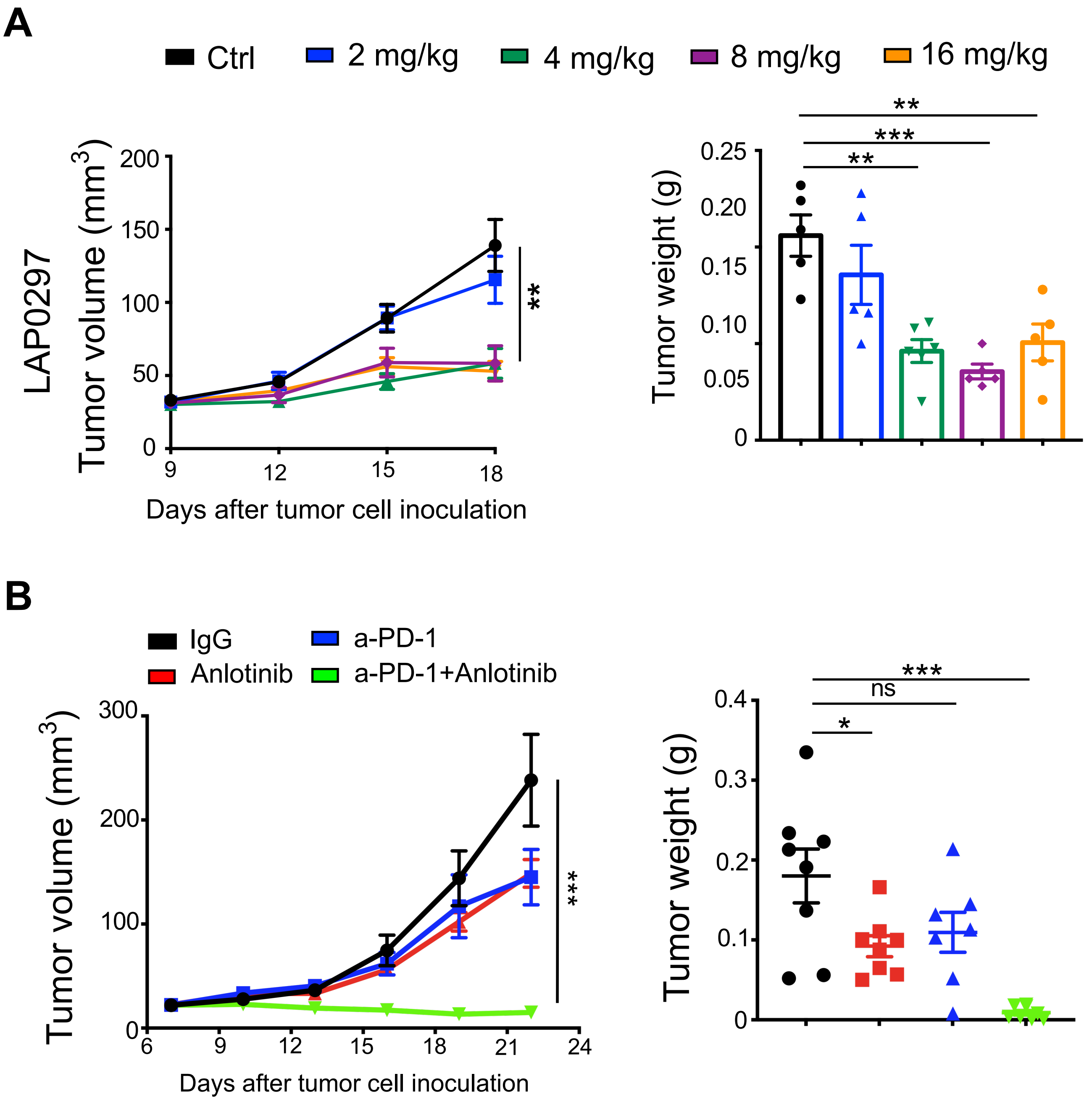
A
Ctrl
2 mg/kg
4 mg/kg
8 mg/kg
16 mg/kg
LAP0297
Tumor volume (mm3)
200
150
100
50
0
9
12
15
18
Days after tumor cell inoculation
**
Tumor weight (g)
0.25
0.20
0.15
0.10
0.05
0
**
***
**
B
IgG
a-PD-1
Anlotinib
a-PD-1+Anlotinib
Tumor volume (mm3)
300
200
100
0
6
9
12
15
18
21
24
Days after tumor cell inoculation
***
Tumor weight (g)
0.4
0.3
0.2
0.1
0
***
ns
*

Fig. S2

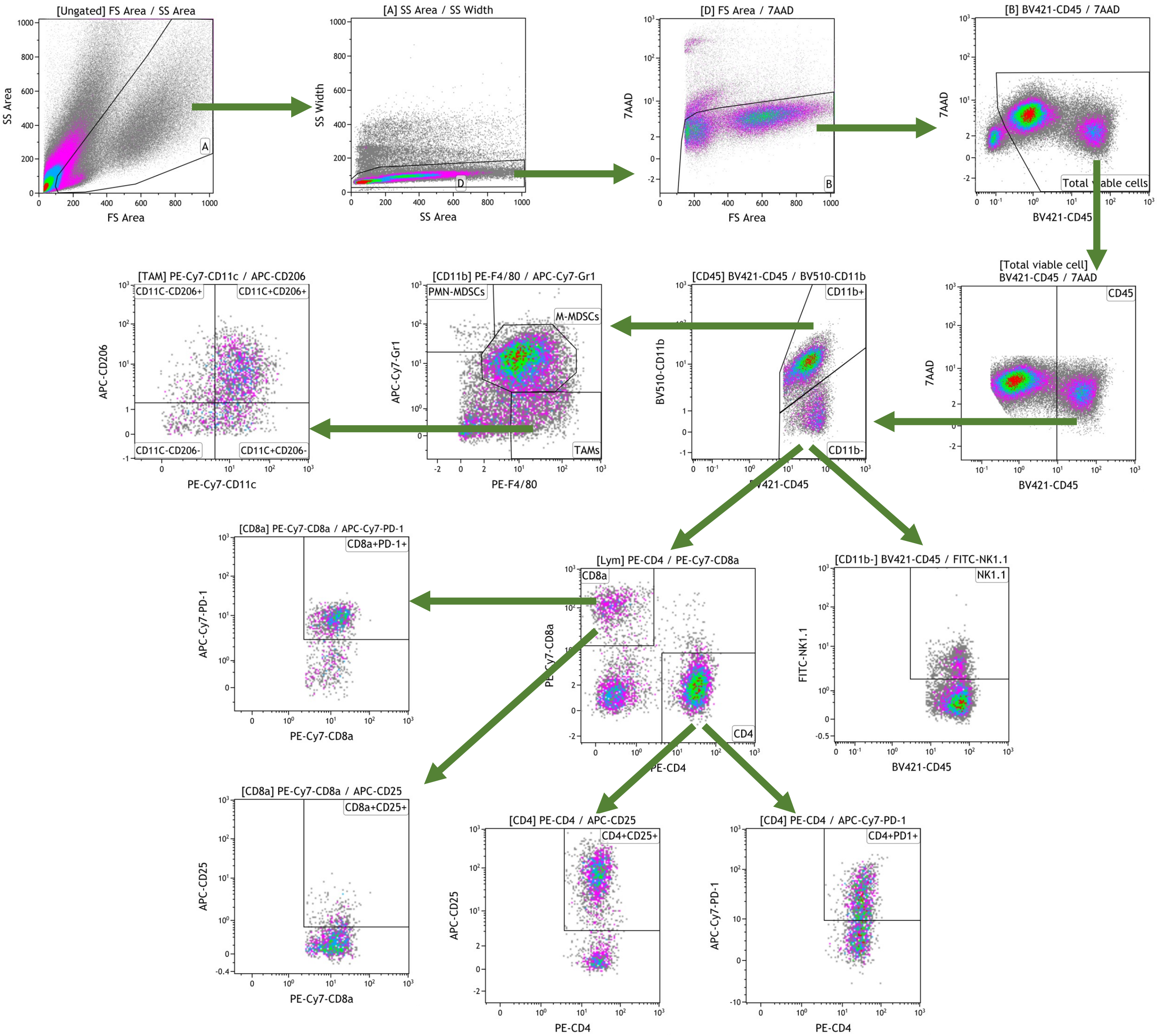
[Ungated] FS Area / SS Area
[A] SS Area / SS Width
[D] FS Area / 7AAD
[B] BV421-CD45 / 7AAD
Total viable cells
[Total viable cell] BV421-CD45 / 7AAD
CD45
[CD45] BV421-CD45 / BV510-CD11b
CD11b+
CD11b-
[CD11b] PE-F4/80 / APC-Cy7-Gr1
PMN-MDSCs
M-MDSCs
TAMs
[TAM] PE-Cy7-CD11c / APC-CD206
CD11C-CD206+
CD11C+CD206+
CD11C-CD206-
CD11C+CD206-
[Lym] PE-CD4 / PE-Cy7-CD8a
CD8a
CD4
[CD11b-] BV421-CD45 / FITC-NK1.1
NK1.1
[CD8a] PE-Cy7-CD8a / APC-Cy7-PD-1
CD8a+PD-1+
[CD8a] PE-Cy7-CD8a / APC-CD25
CD8a+CD25+
[CD4] PE-CD4 / APC-CD25
CD4+CD25+
[CD4] PE-CD4 / APC-Cy7-PD-1
CD4+PD1+

Fig. S3

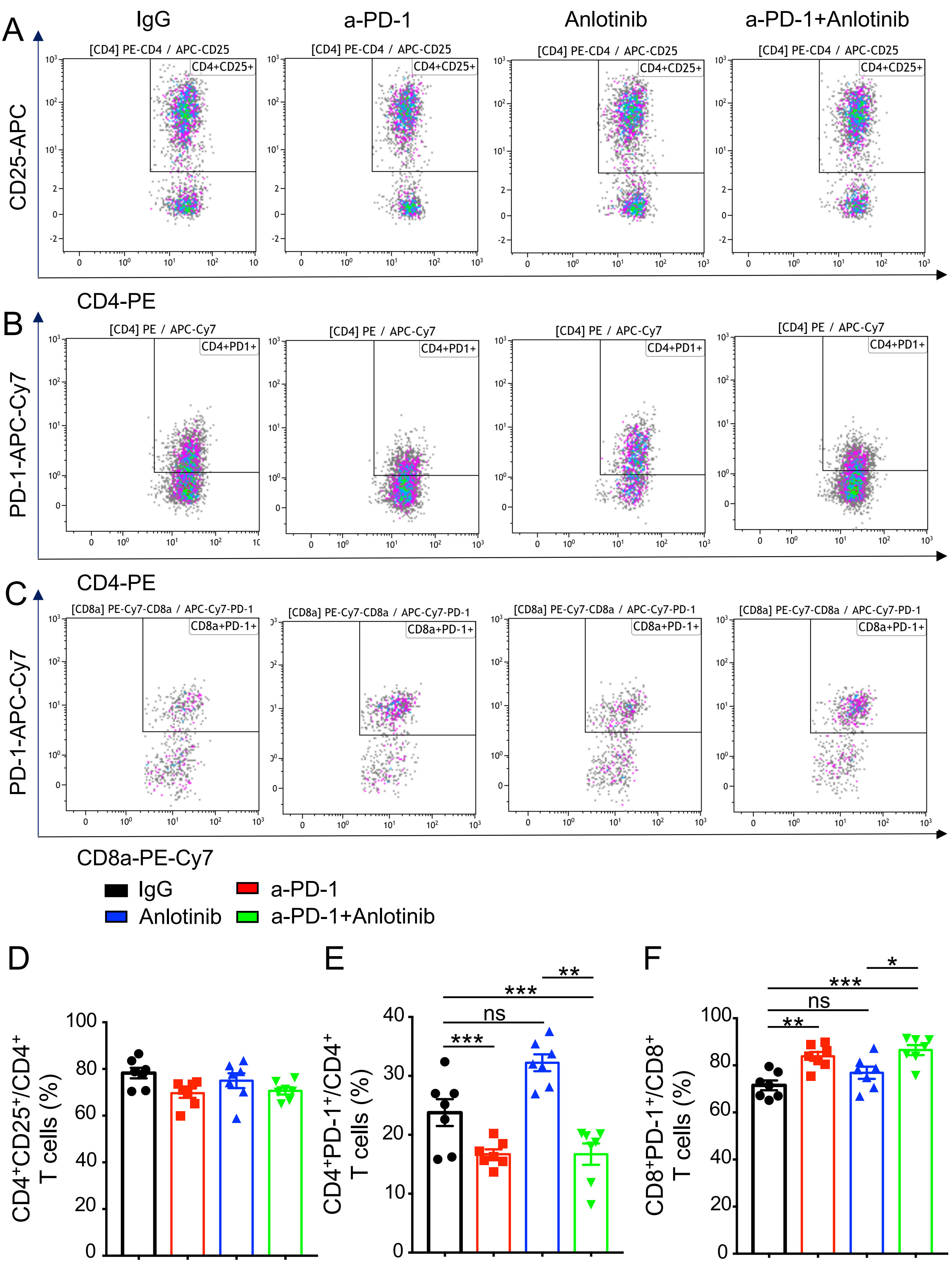
A
IgG
a-PD-1
Anlotinib
a-PD-1+Anlotinib
[CD4] PE-CD4 / APC-CD25
CD4+CD25+
CD25-APC
CD4-PE
B
[CD4] PE / APC-Cy7
CD4+PD1+
PD-1-APC-Cy7
CD4-PE
C
[CD8a] PE-Cy7-CD8a / APC-Cy7-PD-1
CD8a+PD-1+
PD-1-APC-Cy7
CD8a-PE-Cy7
IgG
a-PD-1
Anlotinib
a-PD-1+Anlotinib
D
CD4+CD25+/CD4+ T cells (%)
E
CD4+PD-1+/CD4+ T cells (%)
***
ns
***
**
F
CD8+PD-1+/CD8+ T cells (%)
**
ns
***
*

Fig. S4

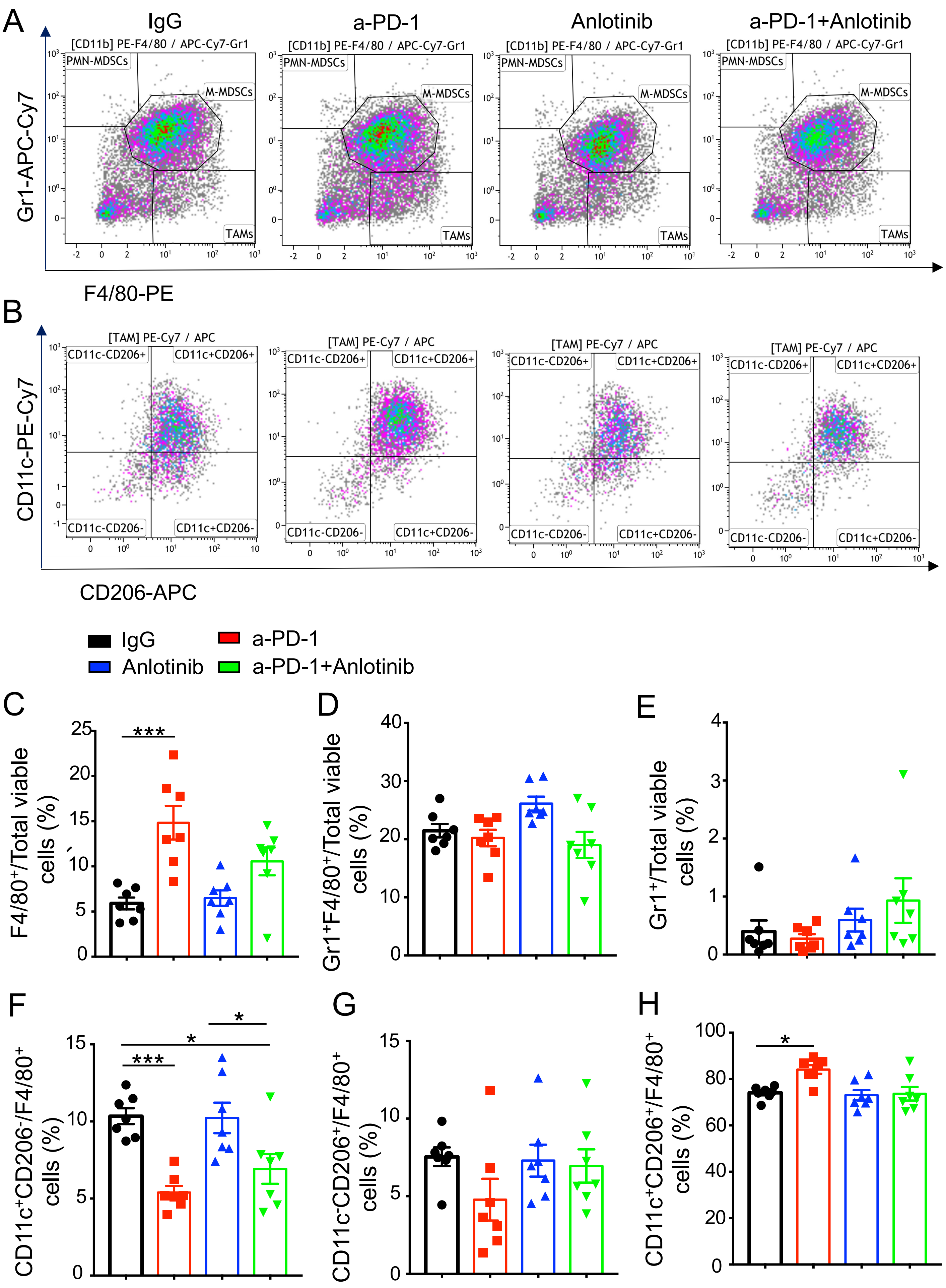
A
IgG
a-PD-1
Anlotinib
a-PD-1+Anlotinib
[CD11b] PE-F4/80 / APC-Cy7-Gr1
PMN-MDSCs
M-MDSCs
TAMs
Gr1-APC-Cy7
F4/80-PE
B
[TAM] PE-Cy7 / APC
CD11c-CD206+
CD11c+CD206+
CD11c-CD206-
CD11c+CD206-
CD11c-PE-Cy7
CD206-APC
IgG
a-PD-1
Anlotinib
a-PD-1+Anlotinib
C
F4/80+/Total viable cells (%)
***
D
Gr1+F4/80+/Total viable cells (%)
E
Gr1+/Total viable cells (%)
F
CD11c+CD206-/F4/80+ cells (%)
***
*
*
G
CD11c-CD206+/F4/80+ cells (%)
H
CD11c+CD206+/F4/80+ cells (%)
*

Fig. S5

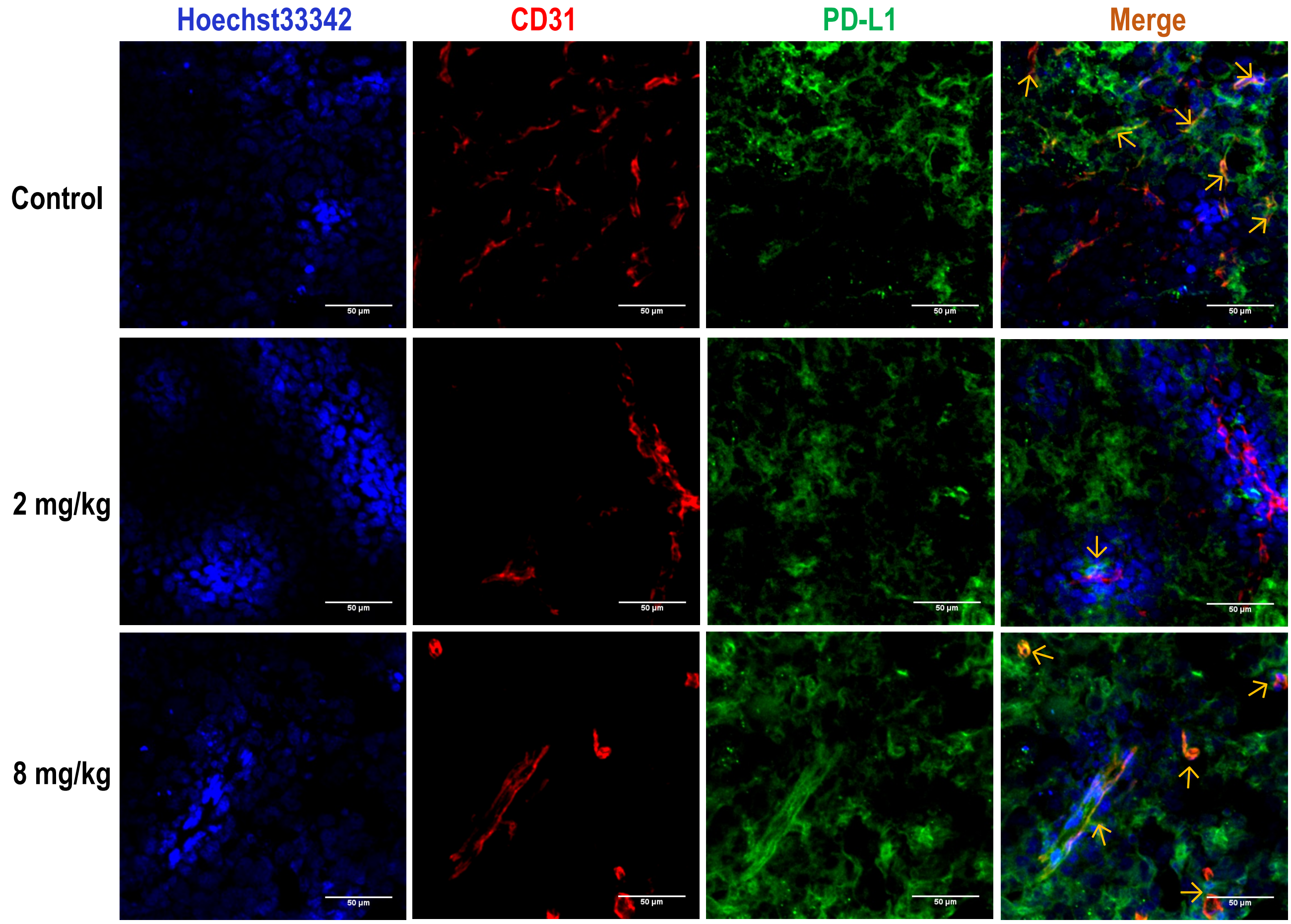

Supplement: Supplementary file 1 [file Presentation_1.pdf]
